# One-Year Tuberculosis Risk in Rheumatoid Arthritis Patients Starting Their First Tumor Necrosis Factor Inhibitor Therapy from 2008 to 2012 in Taiwan: A Nationwide Population-Based Cohort Study

**DOI:** 10.1371/journal.pone.0166339

**Published:** 2016-11-10

**Authors:** Chong-Hong Lim, Ching-Heng Lin, Der-Yuan Chen, Yi-Ming Chen, Wen-Cheng Chao, Tsai-Ling Liao, Hsin-Hua Chen

**Affiliations:** 1 Rheumatology Unit, Department of Internal Medicine, Pulau Pinang General Hospital, Georgetown, Malaysia; 2 Division of Allergy, Immunology and Rheumatology, Department of Internal Medicine, Taichung Veterans General Hospital, Taichung, Taiwan; 3 Department of Medical Research, Taichung Veterans General Hospital, Taichung, Taiwan; 4 School of Medicine, National Yang-Ming University, Taipei, Taiwan; 5 Institute of Biomedical Science and Rong Hsing Research Center for Translational Medicine, Chung-Hsing University, Taichung, Taiwan; 6 School of Medicine, Chung-Shan Medical University, Taichung, Taiwan; 7 Department of Medical Education, Taichung Veterans General Hospital, Taichung, Taiwan; 8 Division of Chest Medicine, Department of Internal Medicine, Taichung Veterans General Hospital, Taichung, Taiwan; 9 Institute of Public Health and Community Medicine Research Center, National Yang-Ming University, Taipei, Taiwan; Mayo Clinic Rochester, UNITED STATES

## Abstract

**Objective:**

To investigate the risk of tuberculosis (TB) among rheumatoid arthritis (RA) patients within 1 year after initiation of tumor necrosis factor inhibitor (TNFi) therapy from 2008 to 2012.

**Methods:**

We used the 2003–2013 Taiwanese National Health Insurance Research Database to identify RA patients who started any RA-related medical therapy from 2008 to 2012. Those who initiated etanercept or adalimumab therapy during 2008–2012 were selected as the TNFi group and those who never received biologic disease-modifying anti-rheumatic drug therapy were identified as the comparison group after excluding the patients who had a history of TB or human immunodeficiency virus infection/acquired immune deficiency syndrome. We used propensity score matching (1:6) for age, sex, and the year of the drug index date to re-select the TNFi group and the non-TNFi controls. After adjusting for potential confounders, hazard ratios (HRs) with 95% confidence intervals (CIs) were calculated to examine the 1-year TB risk in the TNFi group compared with the non-TNFi controls. Subgroup analyses according to the year of treatment initiation and specific TNFi therapy were conducted to assess the trend of 1-year TB risk in TNFi users from 2008 to 2012.

**Results:**

This study identified 5,349 TNFi-treated RA patients and 32,064 matched non-TNFi-treated controls. The 1-year incidence rates of TB were 1,513 per 10^5^ years among the TNFi group and 235 per 10^5^ years among the non-TNFi controls (incidence rate ratio, 6.44; 95% CI, 4.69–8.33). After adjusting for age, gender, disease duration, comoridities, history of TB, and concomitant medications, TNFi users had an increased 1-year TB risk (HR, 7.19; 95% CI, 4.18–12.34) compared with the non-TNFi-treated controls. The 1-year TB risk in TNFi users increased from 2008 to 2011 and deceased in 2012 when the Food and Drug Administration in Taiwan announced the Risk Management Plan for patients scheduled to receive TNFi therapy.

**Conclusion:**

This study showed that the 1-year TB risk in RA patients starting TNFi therapy was significantly higher than that in non-TNFi controls in Taiwan from 2008 to 2012.

## Introduction

Tuberculosis (TB) is an ancient, contagious airborne disease that has been in existence for centuries; currently, the disease is still an alarming global health issue. In 2014, the World Health Organization (WHO) reported 9.6 million incident cases of TB. Not surprisingly, TB mortality remains one of the leading causes of death worldwide, with the estimated mortality of 1.5 million per year [1]. In Taiwan, TB is not uncommon and generates a moderate healthcare burden. The Taiwan Centers for Disease Control reported 11,528 cases of TB (49.4 cases per 100,000 populations) and 609 TB-related deaths in 2013[2].

Rheumatoid arthritis (RA) is a well-established risk factor for TB [3–8]. In Taiwan, the risk of TB development was 2.28-fold higher in RA patients than in the general population [8]. Tumor necrosis factor (TNF) plays a key role in the immunity against TB [9]. In recent years, the use of a TNF inhibitor (TNFi) in RA patients further increased the TB risk [7, 10–12]. Furthermore, prior studies have shown that monoclonal antibodies to TNF, such as infliximab (IFX) and adalimumab (ADA), may drive higher TB risk than TNF receptor blockers such as etanercept (ETN) [10, 13].

The Bureau of National Health Insurance in Taiwan approved the first TNFi ETN for RA patients with inadequate response to traditional disease-modifying anti-rheumatic drugs (DMARDs) in 2003, followed by ADA in April 2007 and golimumab in 2012. IFX and certolizumab were not available in Taiwan. During 2006–2008, the risk of TB was 4.87-fold higher among TNFi users than among non-TNFi users in Taiwan [11]. Therefore, in 2011, the Taiwan Rheumatology Association (TRA) established a Biologics TB Safety Management Working Group [14]. In 2011, this Working Group published a preliminary recommendation for screening of latent TB infection (LTBI) and prophylactic/therapeutic strategies for rheumatic patients who are scheduled for biologics therapy [15]. Since then, more and more rheumatologists began screening LTBI using the tuberculin skin test and quantiferon blood test and administered isoniazid (INH) prophylaxis for screening-positive cases before TNFi use. In April 2012, the Food and Drug Administration (FDA) in Taiwan announced the Risk Management Plan (RMP) for patients scheduled to receive TNFi therapy [16]. In July 2012, the TRA Biologics TB Safety Working Group published a consensus on recommendations for screening and management of TB infection in patients scheduled for TNFi therapy[14].

We had previously found a biphasic emergence of active TB infection in TNFi users [17]. The early development was due to reactivation of LTBI, while the late emergence was more likely to result from new TB exposure [17]. We hypothesized that screening and treatment of LTBI might reduce the risk of early TB development in users of TNFi, with a greater effect for the TNF monoclonal antibody ADA than the TNF receptor blocker ETN. To our knowledge, the drug-specific 1-year TB risk in patients with RA starting TNFi therapy has never been investigated in Taiwan as well as in other countries. Therefore, the aim of this study was to compare the 1-year TB risk between biologic-naive RA patients initiating ETN or ADA therapy from 2008 to 2012 and RA patients who never received biologic treatment during 2003–2013.

## Methods

### Ethics statement

This study was conducted in concordance with the Declaration of Helsinki and was approved by the Institutional Review Board (IRB) of Taichung Veterans General Hospital Taiwan (IRB number: CE14149). All personal details traced were anonymized before analysis; hence, informed consent was not obtained.

### Study design and data source

This study was a retrospective cohort study using the 2003–2013 claim data from the National Health Insurance Research Database (NHIRD) of Taiwan. Single-payer National Health Insurance (NHI) is a mandatory program launched in 1995, which currently covers 99.9% of the Taiwanese comprising 23 million people [18]. The National Health Research Institute managed the NHIRD and released the database for research purpose.

### Study population

We identified all patients with at least three ambulatory visits or one hospital admission with a diagnosis of RA [International Classification of Diseases, 9th Revision, Clinical Modification (ICD-9-CM) Codes 714.0] and concurrent prescription of RA-related medications during 2003–2013 as RA patients. RA-related medications included methotrexate (MTX), sulfasalazine (SSZ), leflunomide (LEF), hydroxychloroquine (HCQ), cyclosporine (CSA), azathioprine (AZA), nonsteroidal anti-inflammatory agents (NSAIDs), corticosteroid (CS), and biologics. Because golimumab was approved in 2012 in Taiwan and the case number of golimumab users in 2012 was relatively small (n = 111), we selected all biologic-naive RA patients who started ETN or ADA therapy from 2008 to 2012 as the TNFi group. We identified RA patients who initiated RA related medical therapy from 2008 to 2012 and have never received biologic therapy during 2003–2013 as the non-TNFi control group. We excluded those who had a diagnosis of TB [ICD-9-CM (010–018)] or human immunodeficiency virus infection/acquired immune deficiency syndrome [ICD-9-CM (042–043)] before the drug index date. For each TNFi user, we randomly selected 6 matched controls from the non-TNFi group using the propensity score matching technique to comprise baseline differences between the TNFi group and the non-TNFi control group. The propensity score was estimated from a multivariable logistic regression medel. We included age, sex, and the year of the index date in the model.

### Index date

The drug index date was the first date of ETN or ADA prescription in the TNFi group and the date of the first non-biologic RA-related medicine prescription in non-TNFi controls. We excluded those who had TB diagnosis [ICD-9-CM (010–018)] within 6 months before the index date.

### Outcome analysis and follow-up

The outcome of this study was the time from the drug index date to the occurrence of TB infection within one year after the drug index date. The censored date was the date of withdrawal from the NHI for any reason within 1 year after the drug index date, 90 days after the last date of drug prescription (ETN or ADA in the study group and non-biologic medication in controls) within 1 year, or 365 days after the drug index date, whichever came first.

### Definition of TB occurrence

TB was identified using ICD-9-CM (010–018) and the prescription of at least two anti-TB drugs within six months of the diagnosis. Anti-TB drug prescriptions included INH, ethambutol, rifampin, pyrazinamide, prothionamide, streptomycin, kanamycin, amikacin, levofloxacin, ofloxacin, moxifloxacin, ciprofloxacin, clarithromycin, and thioridazine. A previous study has validated this method [19].

### Covariates

Other potentially confounding covariates included age (≤65 years, >65 years), gender, disease duration, comorbidities, history of TB, INH prophylaxis, and concomitant medications. Disease duration was defined as the time from the first date of RA diagnosis to the drug index date. Comorbidities were identified using ICD-9-CM codes (Table A in S1 Table) within six months before and after the index date [20]. Except for the periodontitis, these comorbidities need to appear in at least three outpatient clinic visits separately or during any of the hospital admission. Periodontitis was defined as having at least one dental visit with concurrent periodontal treatment other than dental scaling or at least three dental visits with concurrent dental scaling [21]. INH prophylaxis was defined as the prescription of INH but no other anti-TB antibiotics within one month before the index date and within nine months after the index date. Concomitant medications included MTX, SSZ, LEF, HCQ, CSA, AZA, CS, and NSAIDs.

### Statistical analysis

The baseline demographic data were presented as a mean ± standard deviation for continuous variables and as a percentage of patients for categorical variables. The differences were analyzed using Student’s *t*-test for continuous variables and Pearson’s χ^2^ test for categorical variables. The 1-year TB risk of TNFi users compared with controls was shown as incidence rate ratios (IRRs) and adjusted hazard ratios (aHRs) using Cox regression analysis after adjusting for potential confounders, including age, sex, disease duration, history of TB, comorbidities, and concomitant medications, further stratified by the year of treatment initiation. All data were analyzed using statistical software version 9.3 (SAS Institute, Inc., Cary, NC, USA). A P value <0.05 was considered statistically significant.

## Results

This study identified a total of 51,576 RA patients, including 5,638 TNFi users and 45,938 controls. Table B in S1 Table reveals baseline demographic and clinical data among the two groups. Then, we used propensity score matching (1:6) for age, sex, and the year of the drug index date to re-select 5,349 TNFi users and 32,094 controls. Table 1 shows the comparison of baseline demographic and clinical data among the two re-selected groups. The proportions of patients with coronary artery disease, cerebrovascular disease, malignancy, hyperlipidemia, or hypertension were higher in controls than in TNFi users. On the other hand, the percentages of patients with concomitant use of MTX, SSZ, LEF, HCQ, CSA, AZA, or CS were higher in TNFi users than in controls. Patients in the control group had shorter disease duration than that of TNFi users (0.2 ± 1.0 vs. 4.6 ± 2.7 years). The comparison of demographic and clinical characteristics between ETN users and ADA users is shown in Table C in S1 Table. Compared with ETN users, ADA users had longer disease duration and higher proportions of concurrent use of LEF, CSA, AZA, and a higer dose of CS (≥7.5 prednisolone equivalent) and prior use of LEF, CSA, and AZA. On the contrary, a lower proportion of ADA users had concomitant use of SSZ, HCQ, and prior use of SSZ compared with that of ETN users.

**Table 1:** Demographic data and clinical characteristics of patients.

	Non-TNFi	TNFi	
	(*n* = 32,094)	(*n* = 5,349)	P value
**Age, years** (mean ± SD)	55 ± 13	55 ± 13	1
**Age group**			
<65 years	24,318 (75.8)	4,053 (75.8)	
≥65 years	7,776 (24.2)	1,296 (24.2)	
**Gender**			1
Female	25,770 (80.3)	4,295 (80.3)	
Male	6,324 (19.7)	1,054 (19.7)	
**Disease duration, years** (mean ± SD)	0.2 ± 1.0	4.6 ± 2.7	<0.001
**Comorbidities**			
Periodontal disease	4,503 (14.0)	721 (13.5)	0.281
Diabetes mellitus	4,200 (13.1)	479 (9.0)	<0.001
COPD	2,410 (7.5)	411 (7.7)	0.654
Liver disease	1,711 (5.3)	272 (5.1)	0.456
Renal disease	912 (2.8)	131 (2.5)	0.106
Heart failure	531 (1.7)	83 (1.6)	0.583
Coronary artery disease	2,351 (7.3)	258 (4.8)	<0.001
Cerebrovascular disease	1,245 (3.9)	130 (2.4)	<0.001
Malignancy	1,379 (4.3)	97 (1.8)	<0.001
Hypertension	9,523 (29.7)	1,486 (27.8)	0.004
Hyperlipidemia	4,995 (15.6)	469 (8.8)	<0.001
Hyperthyroidism	386 (1.2)	59 (1.1)	0.533
**Isoniazid prophylaxis**	16 (0.1)	230 (4.3)	<0.001
**Concomitant medications**			
Methotrexate	5,414 (16.9)	4,047 (75.7)	<0.001
Sulfasazaline	7,775 (24.2)	2,729 (51.0)	<0.001
Leflunomide	677 (2.1)	1,157 (21.6)	<0.001
Hydroxychloroquine	11,918 (37.1)	2,812 (52.6)	<0.001
Cyclosporine	274 (0.9)	437 (8.2)	<0.001
Azathioprine	335 (1.0)	96 (1.8)	<0.001
Corticosteroid			<0.001
None	19,134 (59.6)	879 (16.4)	
<7.5 mg/day*	6,295 (19.6)	2,524 (47.2)	
≥7.5 mg/day*	6,665 (20.8)	1,946 (36.4)	
NSAIDs	30,160 (94.0)	5,091 (95.2)	<0.001

Abbreviations: TNFi, tumor necrosis factor inhibitor; SD, standard deviation; COPD, chronic obstructive pulmonary disease; NSAIDs, nonsteroidal anti-inflammatory drugs.

*Prednisolone equivalent dose.

As shown in Table 2, a total of 154 TB events were reported within 1 year after the index date; 74 (0.23%) in the control group and 80 (1.50%) in the TNFi group. The 1-year IR of TB was higher in TNFi users then in non-TNFi controls (1,513 per 10^5^ years vs. 235 per 10^5^ years; IRR, 6.44; 95% CI, 4.69–8.33). Compared with the control group, the 1-year IR of TB was higher in ETN users (IRR, 2.93; 95% CI, 1.79–4.81) and ADA users (IRR, 10.69; 95% CI, 7.61–15.03).

**Table 2:** One-year incidence rate of tuberculosis (TB).

	Total Patients	TB Event, N (%)	Person-years	Incidence Rate (/10^5^ years)	IRR (95% CI)
Group					
Non-TNFi	32,094	74 (0.23)	31,481	235	Reference
TNFi	5,349	80 (1.50)	5,287	1,513	6.44 (4.69–8.83)
Etanercept	2,925	20 (0.68)	2,900	690	2.93 (1.79–4.81)
Adalimumab	2,424	60 (2.48)	2,387	2,514	10.69 (7.61–15.03)
Total	37,443	154 (0.41)	36,769	419	-

Abbreviations: TNFi, tumor necrosis factor inhibitor; IRR, incidence rate ratio

**Fig 1:**
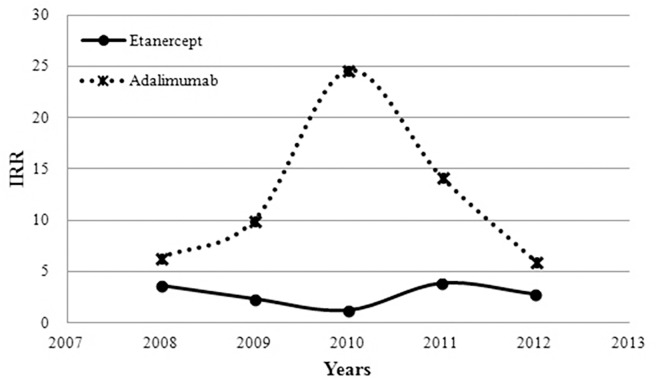
One-year TB incidence rate ratios (IRRs) in rheumatoid arthritis patients treated with etanercept or adalimumab compared with non-TNFi-treated controls.

As shown in Table 3, the 1-year IR of TB in controls peaked in 2008 and was lowest in 2010. However, the 1-year IR of TB in TNFi users peaked in 2010 and then decreased. The IRR of TB in TNFi users compared with controls increased from 4.81 in 2008 to 12.02 in 2010 and then dropped to 4.25 in 2012. The 1-year IRR of TB in ETN users and ADA users compared with non-TNFi controls are shownn in Table D and Table E in S1 Table, respectively. The IRRs of TB in ETN users and ADA users compared with controls are shown in Fig 1. Among ADA users, the 1-year IRR of TB showed a trend of elevation from 2008 to 2010, followed by a trend of decline. However, ETN users showed the opposite trend.

**Table 3:** One-year IRR of tuberculosis according to the year of treatment initiation.

	Non-TNFi						TNFi		
Year	Total	Event (%)	Person-years	IR, /10^5^ years	Total	Event (%)	Person-years	IR, /10^5^ years	IRR (95%CI)
2008	6,396	20 (0.31)	6,331	316	1,066	16 (1.50)	1,054	1,519	4.81 (2.49–9.28)
2009	7,026	18 (0.26)	6,948	259	1,171	17 (1.45)	1,157	1,469	5.67 (2.92–11.00)
2010	5,880	9 (0.15)	5,810	155	980	18 (1.84)	967	1,862	12.02 (5.40–26.75)
2011	6,612	12 (0.18)	6,487	185	1,102	18 (1.63)	1,090	1,651	8.93 (4.30–18.53)
2012	6,180	15 (0.24)	5,905	254	1,030	11 (1.07)	1,019	1,079	4.25 (1.95–9.25)
**Total**	32,094	74 (0.23)	31,481	235	5,349	80 (1.50)	5,287	1,513	6.44 (4.69–8.83)

Abbreviations: TNFi, tumor necrosis factor inhibitor; IRR, Incidence rate ratio.

**Fig 2:**
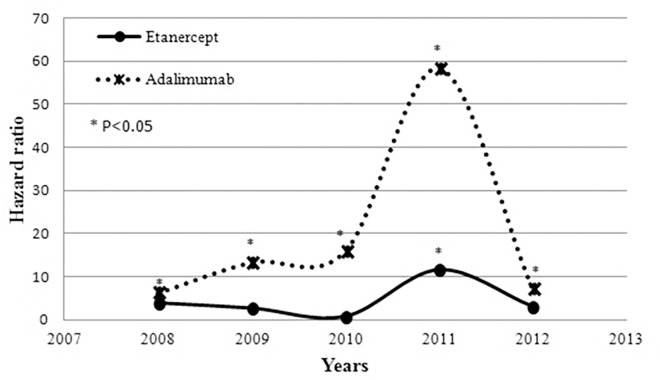
Adjusted hazard ratios of 1-year TB risk in rheumatoid arthritis patients treated with etanercept or adalimumab compared with non-TNFi-treated controls.

After adjusting for potential confounders (Table 4), TNFi users showed a higher risk of TB within one year after treatment initiation compared with non-TNFi controls (HR, 7.19; 95% CI, 4.18–12.34). Other risk factors included age, male gender, the presence of COPD, renal disease or malignancy within six months before and after the index date, and concomitant use of CS with ≥7.5 mg/day prednisolone equivalent dose. Compared with non-TNFi controls, the aHRs (95% CI) of 1-year TB risk for ETN and ADA users were 3.55 (1.85–6.82) and 12.85 (7.27–22.69), respectively. ADA users had a significantly higher risk of TB within one year than ETN users (aHR, 3.62; 95% CI, 2.17–6.03). The aHRs of TB occurrence within one year in ETN users and ADA users compared with non-TNFi controls are shown in Fig 2.

**Table 4:** Multivariable analyses for 1-year tuberculosis risk.

	Univariate analysis	Multivariable analysis
	HR (95% CI)	HR (95% CI)
**Group**		
Non-TNFi	Reference	Reference
TNFi	6.44 (4.70–8.84)	7.19 (4.18–12.34)
**Age**	1.05 (1.04–1.07)	1.04 (1.03–1.06)
**Gender**		
Female	Reference	Reference
Male	2.03 (1.45–2.84)	1.60 (1.13–2.26)
**Disease duration**	1.21 (1.15–1.27)	0.91 (0.84–0.99)
**Isoniazid prophylaxis**	4.02 (1.49–10.84)	1.25 (0.46–3.44)
**Comorbidities**		
Periodontal disease	1.25 (0.82–1.90)	1.20 (0.79–1.83)
Diabetes mellitus	1.49 (0.98–2.26)	1.20 (0.77–1.86)
COPD	3.11 (2.10–4.61)	1.99 (1.32–2.99)
Liver disease	1.66 (0.94–2.93)	1.49 (0.84–2.64)
Renal disease	4.47 (2.70–7.40)	2.69 (1.57–4.62)
Heart failure	4.29 (2.26–8.14)	1.90 (0.96–3.76)
Coronary artery disease	1.76 (1.08–2.88)	1.09 (0.64–1.84)
Cerebrovascular disease	2.44 (1.38–4.31)	1.58 (0.88–2.85)
Malignancy	2.73 (1.60–4.64)	2.31 (1.33–3.99)
Hypertension	1.66 (1.20–2.29)	1.01 (0.70–1.44)
Hyperlipidemia	0.58 (0.34–1.01)	0.53 (0.30–0.94)
Hyperthyroidism	2.79 (1.14–6.79)	3.68 (1.50–9.06)
**Concomitant medications**		
Methotrexate	2.78 (2.03–3.81)	1.14 (0.77–1.69)
Sulfasazaline	1.41 (1.02–1.97)	0.84 (0.59–1.19)
Leflunomide	3.56 (2.30–5.50)	1.15 (0.71–1.86)
Hydroxychloroquine	1.79 (1.30–2.46)	1.28 (0.92–1.78)
Cyclosporine	4.75 (2.69–8.38)	1.77 (0.97–3.24)
Azathioprine	-	-
Steroid		
None	Reference	Reference
<7.5 mg/day*	2.96 (1.94–4.52)	1.46 (0.92–2.33)
≥7.5 mg/day*	4.04 (2.71–6.02)	2.06 (1.32–3.21)
NSAIDs	0.89 (0.47–1.68)	1.00 (0.52–1.93)

Abbreviations: HR, hazard ratio; TNFi, tumor necrosis factor inhibitor; COPD, chronic obstructive pulmonary disease; NSAIDs, nonsteroidal anti-inflammatory drugs.

*Prednisolone equivalent dose.

As shown in Table 5, the aHR of 1-year TB risk in TNFi users compared with non-TNFi controls peaked in 2011 but dropped in 2012. The aHR of 1-year TB risk in ETN users and ADA users compared with non-TNFi controls by year are shown in Table F in S1 Table.

**Table 5:** Multivariable analyses for 1-year tuberculosis risk by year in TNFi users compared with controls.

	Hazard ratio	95% CI	P-value
**2008-Group**	4.85	1.23–19.13	0.024
**2009-Group**	6.22	1.80–21.55	0.004
**2010-Group**	8.84	2.40–32.61	0.001
**2011-Group**	26.42	7.99–87.36	<0.001
**2012-Group**	4.86	1.35–17.45	0.015

Adjusted for patient’s age, gender, disease duration, isoniazid, comorbidities, and concomitant medications. Non-TNFi users were used as controls.

## Discussion

To our knowledge, this study is the first to estimate the 1-year TB infection risk in RA patients initiating TNFi therapy from 2008 to 2012. The decline of 1-year TB incidence rate since 2011 might be explained by the fact that the preliminary recommendation for LTBI screening and prophylactic/therapeutic strategies for RA patients scheduled to start TNFi treatment was published in 2011. After adjusting for potential confounders, the 1-year risk of TB infection in RA patients on TNFi therapy was 7.19-fold higher than that of non-TNFi controls during 2008–2012. This finding was consistent with previous studies demonstrating that TNFi-treated RA patients had an increased risk of TB compared with non-TNFi controls [11, 13, 22, 23]. In contrast to previous studies, this study focused on the 1-year TB risk because the reactivation of LTBI occurred most commonly within one year after TNFi initiation [24, 25]. The magnitude of 1-year TB relative risk (HR, 7.19) in TNFi users compared with non-TNFi controls was higher than that of the overall TB relative risk (HR, 4.87) estimated during 2006–2008 in Taiwan [11]. Because this study estimated only the early (1-year) risk of TB, this finding could be explained by an early emergence of TB infection in TNFi users due to reactivation of LTBI [17]. The aHR of TB infection within one year in TNFi users compared with controls peaked in 2011 (aHR, 26.42) and decreased in 2012 (aHR, 4.86) when the FDA in Taiwan announced the RMP for TB. The physicians’ and patients’ awareness of TNFi-related TB infection risk was believed to increase after the implementation of RMP for TB. Among ADA users, the decrease of HR from 2011 to 2012 was even more prominent (HR 58.38 in 2011 and 7.07 in 2012). This finding may be explained by the hypothesis that LTBI screening/treatment might confer a greater benefit for TNFi therapy that drives higher TB risk. The benefit of LTBI screening/treatment strategy before TNFi therapy has been demonstrated in a study from BIOBADASER national registry, revealing less incidence of TB infection after implementation of the TB prevention strategy [26]. However, because the NHIRD lacked information on individual LTBI screening status, we cannot conclude that RMP implementation may help decrease TB risk based on the results of our study. Regarding the trends of 1-year TB risk in ADA users, the peak moved from 2010 in Fig 1 to 2011 in Fig 2. It is difficult to explain this finding, but adjustment for potential confounders may be one of the causes.

After adjustment for all potential confounders, the 1-year TB risk was significantly higher in ADA users than in ETN users (aHR, 3.62; 95% CI: 2.17–6.03). This finding was comparable to the result of a meta-analysis of randomized controlled trials and registry/cohort studies [10]. The risk of TB infection in ETN users has been demonstrated to be lower than that in patients taking monoclonal antibodies to TNF [13, 23, 27]. However, the NHIRD lacks information on disease activity, which was another potential confounder and might be related to disease duration and immunosuppressive agent use. Given the difference in disease duration and immunosuppressive load between ADA users and ETN users and also between TNFi users and non-TNFi controls, we cannot exclude the possibility that the difference of 1-year TB risk was actually attributed to a difference in disease activity between the two groups.

Consistent with previous studies [28–40], our study showed that older age, male gender, COPD, renal disease, and CS use with ≥7.5 mg/day prednisolone equivalent dose were also risk factors for TB infection.

The strength of this study is the utilization of a population-based cohort to minimize selection bias. However, some limitations must be addressed. First, the various TNFi agents studied in this observation was limited to only ETN and ADA because the newer golimumab was approved in 2012 in Taiwan and only 111 patients were treated with golimumab in 2012. Second, details of some potential confounding factors such as individual socioeconomic status, disease activity, smoking status, family history of TB, and body mass index were not available in the NHIRD. Third, the accuracy of illness diagnoses according to claims data is an issue of concern. However, the accuracy of disease diagnoses had improved after the regular check of the original medical chart by the Bureau of NHI [41]. Furthermore, the non-differential misclassification bias about TB diagnosis in TNFi users and non-TNFi controls may lead to an underestimation of the degree of association between TNFi use and 1-year TB risk. Finally, confounding by indication is a bias frequently observed in an observation study and may generate biased results in this study.

In conclusion, the 1-year TB risk in RA patients starting TNFi therapy was significantly higher than that in non-TNFi controls. Such an increased risk seemed to decline after the establishment of RMP for TB in 2012, especially in ADA users. Other significant risk factors for 1-year TB infection included male gender, age, COPD, renal disease, and concomitant CS use with ≥7.5 mg/day prednisolone equivalent dose. Further study including RA patients initiating TNFi therapy after 2012 is warranted to confirm a consistent trend of decline in 1-year TB risk after the establishment of RMP for TB.

## Supporting Information

S1 Table Supplementary Tables. (DOC)
