# Supplementary material for: One-Year Tuberculosis Risk in Rheumatoid Arthritis Patients Starting Their First Tumor Necrosis Factor Inhibitor Therapy from 2008 to 2012 in Taiwan: A Nationwide Population-Based Cohort Study
Source: PLoS One. 2016 Nov 10;11(11):e0166339. doi: 10.1371/journal.pone.0166339 (PMC5104359; doi:10.1371/journal.pone.0166339)
Supplement: S1 Table — (DOC) [file pone.0166339.s001.doc]

One-Year Tuberculosis Risk in Rheumatoid Arthritis Patients Starting their First Tumor Necrosis Factor Inhibitor Therapy from 2008 to 2012 in Taiwan: A Nationwide Population-Based Cohort Study

**Authors:** Chong-Hong Lim, Ching-Heng Lin, Der-Yuan Chen, Yi-Ming Chen, Wen-Cheng Chao, Tsai-Ling Liao, Hsin-Hua Chen^*^

**Supplementary materials**

Table A. ICD-9-CM of the comorbidities

| Comorbidities | ICD-9-CM |
| --- | --- |
| Periodontal disease | 523.3–523.5 |
| Diabetes mellitus | 250 |
| COPD/ asthma | 490–496, 500–505 and 506.4 |
| Liver diseases | 571.2, 571.4–571.6 (mild)  572.2– 572.8 and 456.0– 456.2 (moderate to severe) |
| Renal diseases | 582– 583 and 585– 588 (moderate to severe) |
| Heart failure | 428 |
| Coronary artery disease | 410 and 412 |
| Cerebrovascular disease | 430– 438 |
| Malignancies | 140–172, 174–195, 200 (any tumor)  204–208 (leukemia)  200–203 (lymphoma)  196– 199 (metastatic) |
| Hypertension | 401.0–401.9 |
| Hyperlipidemia | 272.0– 272.4 |
| Hyperthyroidism | 242.9 |

Abbreviations: COPD, chronic obstructive pulmonary disease; HIV, human immunodeficiency virus; AIDS, acquired immune deficiency syndrome.

Table B. Demographic data and clinical characteristics of patients before matching

|  | Non-TNFi |  | TNFi |  |
| --- | --- | --- | --- | --- |
|  | (*n* =45,938) |  | (*n* =5,638) | P-value |
| **Age, years** (mean ± SD) | 55 ± 15 |  | 55 ± 13 | <0.001 |
| **Age group** |  |  |  |  |
| <65 years | 35,401 (71.5) |  | 4,296 (76.2) |  |
| ≥65 years | 14,137 (28.5) |  | 1,342 (23.8) |  |
| **Gender** |  |  |  | <0.001 |
| Female | 35,368 (71.4) |  | 4,528 (80.3) |  |
| Male | 14,170 (28.6) |  | 1,110 (19.7) |  |
| **Disease duration, years** (mean ± SD) | 0.2 ± 1.0 |  | 4.6 ± 2.7 | <0.001 |
| **Comorbidities** |  |  |  |  |
| Periodontal disease | 6,770 (13.7) |  | 759 (13.5) | 0.672 |
| Diabetes mellitus | 6,463 (13.1) |  | 501 (8.9) |  |
| COPD | 3,990 (8.1) |  | 429 (7.6) | 0.243 |
| Liver disease | 2,580 (5.2) |  | 284 (5.0) | 0.584 |
| Renal disease | 1,612 (3.3) |  | 136 (2.4) | 0.001 |
| Heart failure | 1,028 (2.1) |  | 88 (1.6) | 0.009 |
| Coronary artery disease | 3,941 (8.0) |  | 272 (4.8) | <0.001 |
| Cerebrovascular disease | 2,307 (4.7) |  | 134 (2.4) | <0.001 |
| Malignancy | 2,215 (4.5) |  | 101 (1.8) | <0.001 |
| Hypertension | 15,282 (30.9) |  | 1,539 (27.3) | <0.001 |
| Hyperlipidemia | 7,385 (14.9) |  | 490 (8.7) | <0.001 |
| Hyperthyroidism | 564 (1.1) |  | 62 (1.1) | 0.794 |
| **Isoniazid prophylaxis** | 23 (0.1) |  | 242 (4.3) | <0.001 |
| **Concomitant medications** |  |  |  |  |
| Methotrexate | 8,090 (16.3) |  | 4,264 (75.6) | <0.001 |
| Sulfasazaline | 11,678 (23.6) |  | 2,866 (50.8) | <0.001 |
| Leflunomide | 981 (2.0) |  | 1,206 (21.4) | <0.001 |
| Hydroxychloroquine | 17,703 (35.7) |  | 2,936 (52.1) | <0.001 |
| Cyclosporin | 405 (0.8) |  | 461 (8.2) | <0.001 |
| Azathioprine | 520 (1.1) |  | 102 (1.8) | <0.001 |
| Steroid |  |  |  | <0.001 |
| None | 29,605 (59.8) |  | 930 (16.5) |  |
| <7.5 mg/day* | 9,539 (19.3) |  | 2,682 (47.6) |  |
| ≥7.5mg/day* | 10,394 (21.0) |  | 2,026 (35.9) |  |
| NSAIDs | 46,590 (94.1) |  | 5,363 (95.1) | 0.001 |

Abbreviations: TNFi, tumor necrosis factor inhibitor; SD, standard deviation; COPD, chronic obstructive pulmonary disease; NSAIDs, nonsteroidal anti-inflammatory drugs.

*Prednisolone equivalent dose.

Table C. Comparison of demographic data and clinical characteristics between rheumatoid arthritis patients treated with etanercept and those treated with adalimumab

|  | Etanercept |  | Adalimumab |  |
| --- | --- | --- | --- | --- |
|  | (*n* =2,925) |  | (*n* =2,424) | P-value |
| **Age, years** (mean ± SD) | 54 ± 14 |  | 54 ± 14 | 0.184 |
| **Age group** |  |  |  | 0.184 |
| <65 years | 2,341 (75.3) |  | 1,978 (76.8) |  |
| ≥65 years | 769 (24.7) |  | 598 (23.2) |  |
| **Gender** |  |  |  | 0.726 |
| Female | 2,501 (80.4) |  | 2,062 (80.0) |  |
| Male | 609 (19.6) |  | 514 (20.0) |  |
| **Disease duration, years** (mean ± SD) | 4.4 ± 2.7 |  | 4.7 ± 2.6 | <0.001 |
| **Comorbidities** |  |  |  |  |
| Periodontal disease | 415 (13.3) |  | 353 (13.7) | 0.693 |
| Diabetes mellitus | 6,584 (13.1) |  | 258 (10.0) | 0.006 |
| COPD | 239 (7.6) |  | 198 (7.6) | 0.998 |
| Liver disease | 153 (4.9) |  | 134 (5.2) | 0.628 |
| Renal disease | 78 (2.5) |  | 61 (2.3) | 0.733 |
| Heart failure | 51 (1.6) |  | 40 (1.5) | 0.794 |
| Coronary artery disease | 159 (5.1) |  | 119 (4.6) | 0.390 |
| Cerebrovascular disease | 68 (2.1) |  | 68 (2.6) | 0.265 |
| Malignancy | 57 (1.8) |  | 45 (1.8) | 0.981 |
| Hypertension | 851 (27.3) |  | 699 (27.1) | 0.847 |
| Hyperlipidemia | 259 (8.3) |  | 233 (9.0) | 0.338 |
| Hyperthyroidism | 31 (1.0) |  | 32 (1.2) | 0.378 |
| **Concomitant medications** |  |  |  |  |
| Methotrexate | 2,380 (76.5) |  | 1,918 (74.4) | 0.070 |
| Sulfasazaline | 1,632 (52.4) |  | 1,264 (49.0) | 0.010 |
| Leflunomide | 539 (17.3) |  | 676 (26.2) | 0.026 |
| Hydroxychloroquine | 1,660 (53.3) |  | 1,299 (50.4) | <0.001 |
| Cyclosporine | 207 (6.6) |  | 259 (10.0) | <0.001 |
| Azathioprine | 40 (1.2) |  | 64 (2.4) | <0.001 |
| Corticosteroid |  |  |  | <0.001 |
| None | 518 (16.6) |  | 416 (16.1) |  |
| <7.5 mg/day* | 1,565 (50.3) |  | 1,136 (44.1) |  |
| ≥7.5 mg/day* | 1,027 (33.0) |  | 1,024 (39.7) |  |
| NSAIDs | 2,970 (95.5) |  | 2,438 (94.6) | 0.136 |
| **Prior medications** |  |  |  |  |
| Methotrexate | 2,053 (66.0) |  | 1,729 (67.1) | 0.378 |
| Sulfasazaline | 1,803 (57.9) |  | 1,442 (55.9) | 0.130 |
| Leflunomide | 581 (18.6) |  | 646 (25.0) | <0.001 |
| Hydroxychloroquine | 1,739 (55.9) |  | 1,454 (56.4) | 0.689 |
| Cyclosporine | 324 (10.4) |  | 316 (12.2) | 0.028 |
| Azathioprine | 54 (1.7) |  | 69 (2.6) | 0.015 |
| Corticosteroid | 2,602 (83.6) |  | 2,143 (83.1) | 0.631 |
| NSAIDs | 2,932 (94.2) |  | 2,415 (93.7) | 0.403 |

Abbreviations: COPD, Chronic obstructive pulmonary disease; NSAIDs, nonsteroidal anti-inflammatory drugs.

*Prednisolone equivalent dose.

Table D. Comparison of 1-year incidence rate of tuberculosis according to the year of treatment initiation between etanercept users and non-TNFi controls

|  | Non-TNFi | | | |  | Etanercept | | | |  |  |
| --- | --- | --- | --- | --- | --- | --- | --- | --- | --- | --- | --- |
| Year | Total | Event (%) | Person-years | IR, /10^5^ years |  | Total | Event (%) | Person-years | IR, /10^5^ years |  | IRR (95% CI) |
| 2008 | 6,396 | 20 (0.31) | 6,331 | 316 |  | 612 | 7 (1.14) | 606 | 1,155 |  | 3.66 (1.55–8.65) |
| 2009 | 7,026 | 18 (0.26) | 6,948 | 259 |  | 660 | 4 (0.61) | 655 | 610 |  | 2.36 (0.80–6.96) |
| 2010 | 5,880 | 9 (0.15) | 5,810 | 155 |  | 525 | 1 (0.19) | 521 | 192 |  | 1.24 (0.16–9.78) |
| 2011 | 6,612 | 12 (0.18) | 6,487 | 185 |  | 564 | 4 (0.71) | 558 | 717 |  | 3.88 (1.25–12.02) |
| 2012 | 6,180 | 15 (0.24) | 5,905 | 254 |  | 564 | 4 (0.71) | 560 | 715 |  | 2.81 (0.93–8.48) |
| **Total** | 32,094 | 74 (0.23) | 31,481 | 235 |  | 2,925 | 20 (0.68) | 2,900 | 690 |  | 2.93 (1.79–4.81) |

Abbreviations: TNFi, tumor necrosis factor inhibitor; IR, incidence rate; IRR, incidence rate ratio.

Table E. Comparison of 1-year incidence rate of tuberculosis according to the year of treatment initiation between adalimumab users and non-TNFi control

|  | Non-TNFi | | | |  | Adalimumab | | | |  |  |
| --- | --- | --- | --- | --- | --- | --- | --- | --- | --- | --- | --- |
| Year | Total | Event (%) | Person-years | IR, /10^5^ years |  | Total | Event (%) | Person-years | IR, /10^5^ years |  | IRR (95% CI) |
| 2008 | 6,396 | 20 (0.31) | 6,331 | 316 |  | 454 | 9 (1.98) | 447 | 2,011 |  | 6.37 (2.90–13.98) |
| 2009 | 7,026 | 18 (0.26) | 6,948 | 259 |  | 511 | 13 (2.54) | 502 | 2,590 |  | 10.00 (4.90–20.40) |
| 2010 | 5,880 | 9 (0.15) | 5,810 | 155 |  | 455 | 17 (3.74) | 446 | 3,815 |  | 24.63 (10.98–55.25) |
| 2011 | 6,612 | 12 (0.18) | 6,487 | 185 |  | 538 | 14 (2.60) | 532 | 2,631 |  | 14.22 (6.58–30.75) |
| 2012 | 6,180 | 15 (0.24) | 5,905 | 254 |  | 466 | 7 (1.50) | 460 | 1,523 |  | 6.00 (2.44–14.71) |
| **Total** | 32,094 | 74 (0.23) | 31,481 | 235 |  | 2,424 | 60 (2.48) | 2,387 | 2,514 |  | 10.69 (7.61–15.03) |

Abbreviations: TNFi, tumor necrosis factor inhibitor; IR, incidence rate; IRR, incidence rate ratio.

Table F. Multivariable analyses for 1-year tuberculosis risk by year.

|  | Hazard ratio | (95% CI) | P-value |
| --- | --- | --- | --- |
| **2008-Group** |  |  |  |
| Etanercept | 3.92 | (0.90–17.13) | 0.069 |
| Adalimumab | 6.18 | (1.44–26.58) | 0.014 |
| **2009-Group** |  |  |  |
| Etanercept | 2.70 | (0.61–12.00) | 0.192 |
| Adalimumab | 13.33 | (3.60–49.30) | <0.001 |
| **2010-Group** |  |  |  |
| Etanercept | 0.76 | (0.07–8.22) | 0.825 |
| Adalimumab | 15.96 | (4.23–60.19) | <0.001 |
| **2011-Group** |  |  |  |
| Etanercept | 11.69 | (2.76–49.56) | 0.001 |
| Adalimumab | 58.38 | (16.42–207.52) | <0.001 |
| **2012-Group** |  |  |  |
| Etanercept | 3.14 | (0.69–14.33) | 0.139 |
| Adalimumab | 7.07 | (1.80–27.86) | 0.005 |

Adjusted for patient’s age, gender, disease duration, isoniazid, comorbidities, and concomitant medications.
